# Smac mimetic suppresses tunicamycin-induced apoptosis via resolution of ER stress

**DOI:** 10.1038/s41419-019-1381-z

**Published:** 2019-02-15

**Authors:** Behnaz Ahangarian Abhari, Nicole McCarthy, Marie Le Berre, Michelle Kilcoyne, Lokesh Joshi, Patrizia Agostinis, Simone Fulda

**Affiliations:** 1 Institute for Experimental Cancer Research in Pediatrics, Goethe-University Frankfurt, Komturstrasse 3a, 60528 Frankfurt, Germany; 2 Glycoscience Group, National University of Ireland, Galway, Ireland; 3 Cell Death Research and Therapy Unit, Department of Cellular and Molecular Medicine, KU Leuven, 3000 Leuven, Belgium; 4 German Cancer Consortium (DKTK), Partner Site Frankfurt, Germany; 5 German Cancer Research Center (DKFZ), Heidelberg, Germany

## Abstract

Since Inhibitor of Apoptosis (IAP) proteins have been implicated in cellular adaptation to endoplasmic reticulum (ER) stress, we investigated the regulation of ER stress-induced apoptosis by small-molecule second mitochondria-derived activator of caspase (Smac) mimetics that antagonize IAP proteins. Here, we discover that Smac mimetic suppresses tunicamycin (TM)-induced apoptosis via resolution of the unfolded protein response (UPR) and ER stress. Smac mimetics such as BV6 selectively inhibit apoptosis triggered by pharmacological or genetic inhibition of protein N-glycosylation using TM or knockdown of DPAGT1, the enzyme that catalyzes the first step of protein N-glycosylation. In contrast, BV6 does not rescue cell death induced by other typical ER stressors (i.e., thapsigargin (TG), dithiothreitol, brefeldin A, bortezomib, or 2-deoxyglucose). The protection from TM-triggered apoptosis is found for structurally different Smac mimetics and for genetic knockdown of cellular IAP (cIAP) proteins in several cancer types, underlining the broader relevance. Interestingly, lectin microarray profiling reveals that BV6 counteracts TM-imposed inhibition of protein glycosylation. BV6 consistently abolishes TM-stimulated accumulation of ER stress markers such as glucose-regulated protein 78 (GRP78) and C/EBP homologous protein (CHOP) and reduces protein kinase RNA-like ER kinase (PERK) phosphorylation and X box-binding protein 1 (XBP1) splicing upon TM treatment. BV6-stimulated activation of nuclear factor-κB (NF-κB) contributes to the resolution of ER stress, since NF-κB inhibition by overexpression of dominant-negative IκBα superrepressor counteracts the suppression of TM-stimulated transcriptional activation of CHOP and GRP78 by BV6. Thus, our study is the first to show that Smac mimetic protects from TM-triggered apoptosis by resolving the UPR and ER stress. This provides new insights into the regulation of cellular stress responses by Smac mimetics.

## Introduction

The ER is the site of synthesis, folding, and post-translational modification of secretory and membrane-bound proteins^1^. Conditions that disturb protein folding in the ER cause ER stress and activate a set of signaling pathways collectively termed the UPR^1^. In mammalian cells, ER stress is sensed by three major ER-resident transmembrane proteins, PERK, inositol-requiring enzyme-1 (IRE1), and activating transcription factor 6 (ATF6)^1^. The ER luminal domains of PERK, IRE1, and ATF6 interact with the ER chaperone GRP78/immunoglobulin heavy chain-binding protein (GRP78/BiP). Upon accumulation of unfolded proteins, GRP78 dissociates from these molecules, allowing activation of their signaling functions^2^. Activation of the UPR induces an adaptive response in which the cell attempts to overcome the accumulation of misfolded proteins via translational inhibition, elevated protein degradation, and increased levels of ER chaperones including GRP78, which consequently increases the protein-folding capacity of the ER^3^. Under excessive ER stress, however, persistent accumulation of misfolded proteins and prolonged activation of UPR promotes cell death typically via apoptosis^1^. Signaling to apoptosis in response to severe ER stress is mainly coordinated by the apoptotic PERK-eIF2α-ATF4 arm of the UPR through transcriptional activation of the proapoptotic transcription factor CHOP.

IAP proteins, for example, cIAP1, cIAP2, and X-linked IAP (XIAP), play a key role in the regulation of cell death and survival signaling and are aberrantly expressed in many human cancers^4^. Therapeutic strategies to antagonize IAP proteins involve small-molecule inhibitors that mimic the amino terminus of Smac, an endogenous antagonist of IAP proteins^4^. BV6 represents one of these Smac mimetics that binds to and neutralizes XIAP, cIAP1, and cIAP2^5^. Besides preventing the interaction of XIAP with caspases, Smac mimetics stimulate autoubiquitination of cIAP1 and cIAP2 followed by their proteasomal degradation^5,6^. This leads to activation of the transcription factor NF-κB, expression of NF-κB target genes such as tumor necrosis factor α (TNFα) and TNFα-dependent cell death^5,6^. As cIAP proteins constitutively trigger proteasomal degradation of NF-κB-inducing kinase (NIK) via their E3 ligase activity^5,6^, Smac mimetics engage non-canonical NF-κB signaling. Since NIK mediates a cross-talk between non-canonical and canonical NF-κB signaling^7^, treatment with Smac mimetics can also result in activation of the canonical NF-κB pathway. As IAP proteins have been implicated in cellular adaptation to ER stress^8–10^, in this current study we investigated the regulation of ER stress-induced apoptosis by small-molecule Smac mimetics.

## Results

### Smac mimetics rescue cancer cells from TM-induced apoptosis and loss of clonogenic survival

**Fig. 1:**
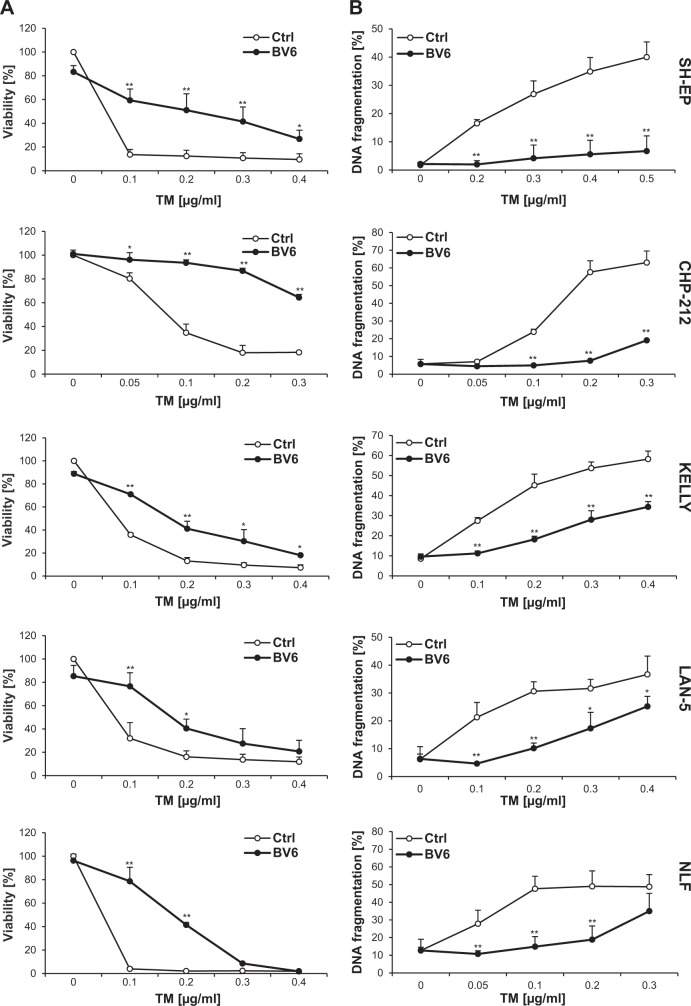

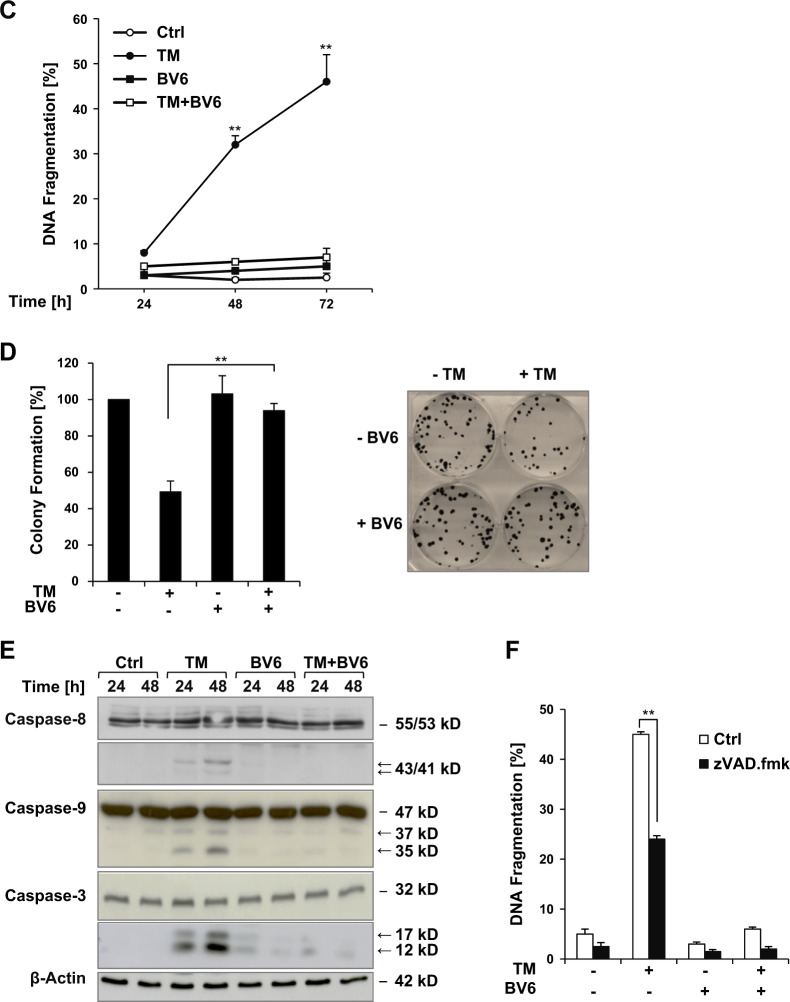
Smac mimetics rescue neuroblastoma cells from TM-induced apoptosis and loss of clonogenic survival. **a**, **b** Neuroblastoma cells were treated for 72 h with indicated concentrations of TM and/or BV6 (SH-EP, LAN-5, KELLY: 4 µM; CHP-212, NLF: 5 µM). Cell viability was determined by MTT assay and is expressed as the percentage of untreated controls (**a**). Apoptosis was determined by flow cytometric analysis of DNA fragmentation of PI-stained nuclei (**b**). **c** SH-EP cells were treated for indicated times with 0.4 µg/ml TM and/or 4 µM BV6. Apoptosis was determined by flow cytometric analysis of DNA fragmentation of PI-stained nuclei. **d** SH-EP cells were treated for 48 h with 0.4 µg/ml TM and/or 4 µM BV6 and colony formation was assessed as described in Materials and methods. The percentage of colony formation compared to untreated control (left panel) and one representative experiment (right panel) are shown. **e** SH-EP cells were treated for indicated times with 0.4 µg/ml TM and/or 4 µM BV6. Caspase activation was analyzed by Western blotting, cleavage fragments are indicated by arrows. β-Actin was used as a loading control. **f** SH-EP cells were treated for 72 h with 0.4 µg/ml TM and/or 4 µM BV6 in the presence or absence of 40 µM zVAD.fmk. Apoptosis was determined by flow cytometric analysis of DNA fragmentation of PI-stained nuclei. **a–d**, **f** Mean ± SEM of three independent experiments performed in triplicate are shown; *P < 0.05; **P < 0.001 comparing samples treated with the combination vs. those treated with TM alone (**a–d**) or comparing samples in the presence or absence of 40 µM zVAD.fmk (**f**). TM, tunicamycin; MTT, 3-(4,5-dimethylthiazol-2-yl)-2,5-diphenyltetrazolium bromide; PI, propidium iodide

To investigate regulation of ER stress-induced cell death by Smac mimetics, we used the nucleoside antibiotic TM as a prototypic ER stress inducer, which inhibits N-linked glycosylation of proteins in the ER, and the Smac mimetic BV6 that antagonizes XIAP, cIAP1, and cIAP2^5^. Unexpectedly, we found that addition of BV6 significantly attenuated TM-induced loss of cell viability in a panel of five neuroblastoma cell lines (Fig. 1a). Similarly, BV6 significantly reduced TM-induced DNA fragmentation, a characteristic feature of apoptotic cell death (Fig. 1b). Kinetic analysis revealed that BV6 protected against TM-induced apoptosis over an extended period of time up to at least 72 h (Fig. 1c). In addition, BV6 significantly inhibited TM-induced loss of colony formation (Fig. 1d), demonstrating that it rescues long-term survival. Monitoring of caspase activation as an additional parameter of apoptosis showed that BV6 inhibited TM-triggered cleavage of caspase-3, -8, and -9 into active fragments (Fig. 1e, Supplementary Figure 1A). The broad-range caspase inhibitor zVAD.fmk significantly reduced TM-induced DNA fragmentation (Fig. 1f, Supplementary Figure 1B), emphasizing caspase-dependent apoptosis.

**Fig. 2:**
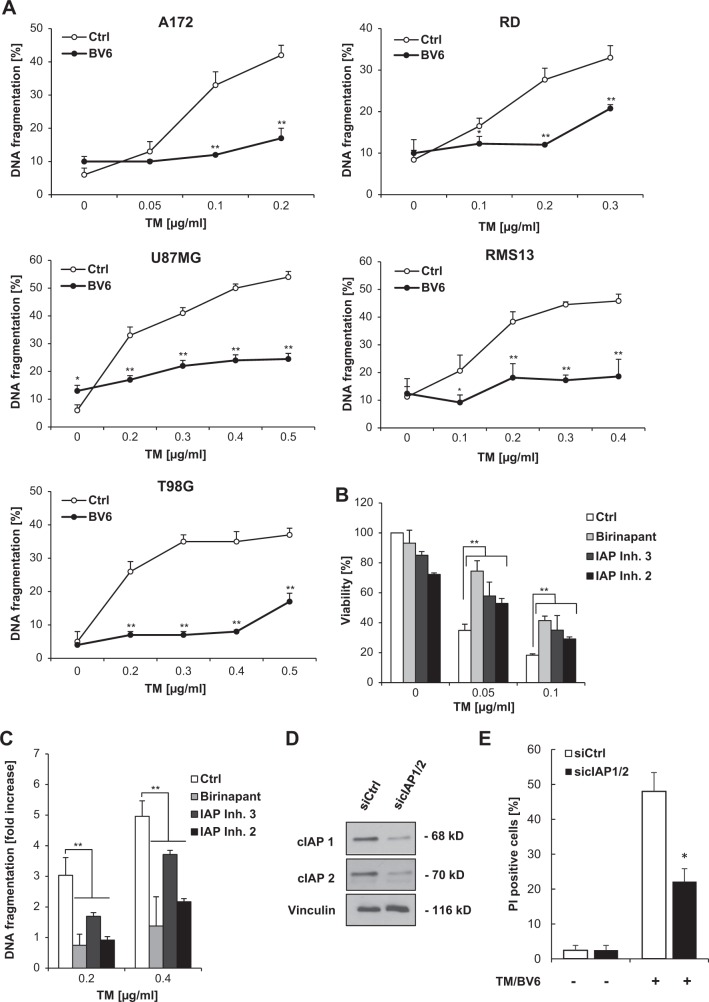
Different Smac mimetics or cIAP1/2 silencing rescue cancer cells from TM-induced cell death. **a** Glioblastoma (A172, U87MG, T98G) and rhabdomyosarcoma (RD, RMS13) cells were treated for 72 h with indicated concentrations of TM and/or BV6 (A172: 2 µM; T98G, RD, RMS13: 3 µM; U87MG: 4 µM). Apoptosis was determined by flow cytometric analysis of DNA fragmentation of PI-stained nuclei. Mean ± SEM of three independent experiments performed in triplicate are shown; *P < 0.05; **P < 0.01. **b** SH-EP cells were treated for 72 h with indicated concentrations of TM and/or different Smac mimetics (birinapant: 30 µM; IAP inhibitor 3: 40 µM; IAP inhibitor 2: 20 µM). Cell viability was determined by MTT assay and is expressed as the percentage of untreated controls. Mean ± SEM values of three independent experiments performed in triplicate are shown; **P < 0.01. **c** SH-EP cells were treated for 72 h with indicated concentrations of TM and/or different Smac mimetics (birinapant: 60 µM; IAP inhibitor 3: 60 µM; IAP inhibitor 2: 60 µM). Apoptosis was determined by flow cytometric analysis of DNA fragmentation of PI-stained nuclei. DNA fragmentation of PI-stained nuclei values was normalized to control values for each condition and fold increase in DNA fragmentation is shown with mean ± SEM values of three independent experiments performed in triplicate; *P < 0.05; **P < 0.01. **d**, **e** SH-EP cells were transiently transfected with siRNAs against cIAP1 and cIAP2 or with control siRNA. cIAP1 and cIAP2 expression was analyzed by Western blotting (**d**), cell death was measured by PI staining and flow cytometry after treatment with 0.1 µg/ml TM and 4 µM BV6 for 48 h (**e**). Mean ± SEM of three independent experiments performed in triplicate are shown; *P < 0.05. TM, tunicamycin; MTT, 3-(4,5-dimethylthiazol-2-yl)-2,5-diphenyltetrazolium bromide; PI, propidium iodide; siRNA, small interfering RNA; cIAP, cellular inhibitor of apoptosis

To ensure that the observed survival effects of BV6 are of broader relevance, we extended our study to other cancer types and Smac mimetics. Similarly, BV6 significantly protected several glioblastoma and rhabdomyosarcoma cell lines against TM-induced apoptosis (Fig. 2a). Moreover, different Smac mimetics inhibited TM-triggered loss of cell viability and apoptosis (Fig. 2b, c).

Since Smac mimetics such as BV6 are described to stimulate autoubiquitination and subsequent proteasomal degradation and depletion of cIAP1 and cIAP2^5^, we then asked whether depletion of cIAP proteins mimics the antiapoptotic function of BV6. To address this question, we simultaneously knocked down cIAP1 and cIAP2 by small interfering RNA (siRNA). Efficient silencing cIAP1 and cIAP2 was controlled by Western blotting (Fig. 2d). Notably, combined silencing of both cIAP1 and cIAP2 significantly rescued TM-induced cell death (Fig. 2e) similar to the protection conferred by BV6 (Fig. 1a–d). This indicates that the BV6-mediated depletion of cIAP proteins is relevant for its protection from TM-induced apoptosis.

### BV6 selectively protects from ER stress-induced apoptosis caused by inhibition of N-linked protein glycosylation

**Fig. 3:**
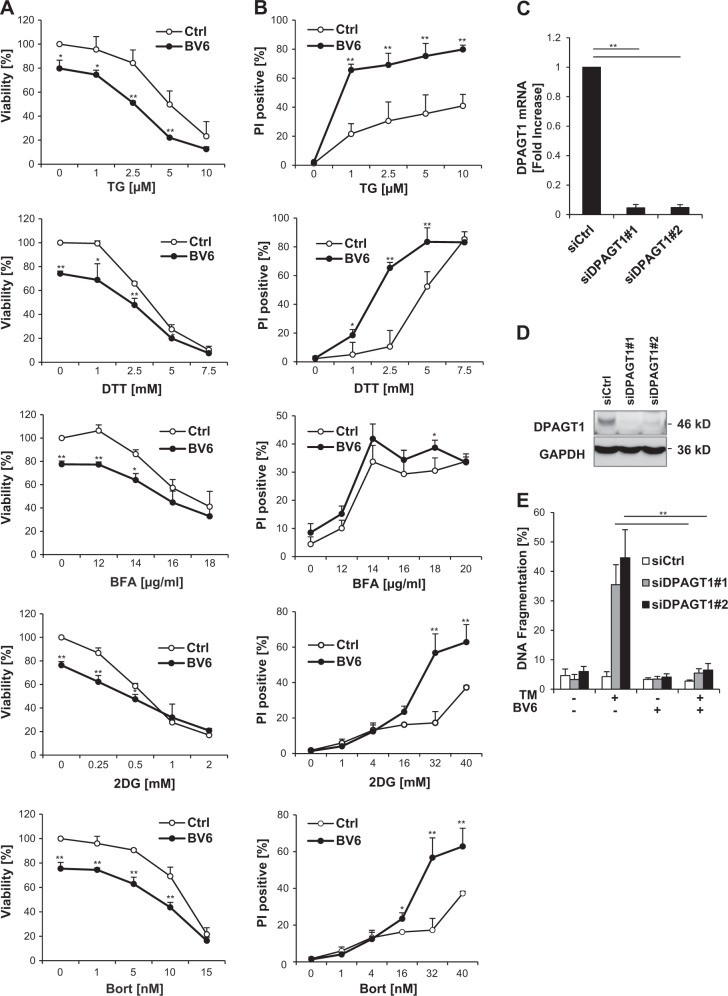
BV6 selectively protects from ER stress-induced apoptosis caused by inhibition of N-linked protein glycosylation. **a**, **b** SH-EP cells were treated for 72 h with indicated concentrations of ER stress inducers and/or 5 µM BV6. Cell viability was determined by MTT assay and is expressed as the percentage of untreated controls (**a**). Cell death was determined by fluorescence-based microscope analysis of PI uptake using Hoechst 33342 and PI double-staining (**b**). Mean ± SEM values of three independent experiments performed in triplicate are shown; *P < 0.05; **P < 0.001 comparing samples treated with the combination vs. those treated with ER stress inducers alone. **c–e** SH-EP cells were transiently transfected with siRNA sequence targeting DPAGT1 (siDPAGT1) or control siRNA (siCtrl). DPAGT1 mRNA levels were analyzed by quantitative RT-PCR and fold increase in DPAGT1 mRNA levels is shown with mean ± SEM values of three independent experiments; **P < 0.001 (**c**). DPAGT1 protein levels were assessed by Western blotting (**d**). Cells were treated with 0.05 µg/ml TM and/or 5 µM BV6 and apoptosis was determined after treatment for 72 h by flow cytometric analysis of DNA fragmentation of PI-stained nuclei (**e**). ER, endoplasmic reticulum; TM, tunicamycin; MTT, 3-(4,5-dimethylthiazol-2-yl)-2,5-diphenyltetrazolium bromide; PI, propidium iodide; siRNA, small interfering RNA; mRNA, messenger RNA; RT-PCR, reverse transcription-PCR

Next, we extended our study to additional ER stress inducers with distinct modes of primary action. In contrast to TM, BV6 failed to rescue cell death in response to TG, a sarcoplasmic/ER calcium ATPase (SERCA) pump inhibitor that depletes ER Ca^2+^ store^11^ (Fig. 3a, b), even though both TM and TG triggered the UPR and ER stress as indicated by GRP78 upregulation and PERK phosphorylation (Supplementary Figure 2) at concentrations that caused a comparable percentage of cell death (Figs. 1b, 3b). Furthermore, BV6 failed to protect from several other ER stress inducers including dithiothreitol (which causes protein misfolding by disrupting disulfide bonds^12^), brefeldin A (which inhibits transport from the ER to the Golgi apparatus^13^), bortezomib (which inhibits proteasomal degradation^14^), and 2-deoxyglucose (which inhibits glycolysis and protein glycosylation^15^) (Fig. 3a, b).

Since TM triggers ER stress by blocking N-linked protein glycosylation in the ER via inhibition of dolichyl-phosphate *N*-acetylglucosamine phosphotransferase 1 (DPAGT1), the enzyme that catalyzes the first step of N-glycosylation^16^, we hypothesized that BV6 protects in particular against ER stress caused by inhibition of N-linked glycosylation. To test this hypothesis, we employed a genetic approach to silence DPAGT1 using two distinct siRNA sequences, which profoundly suppressed messenger RNA (mRNA) and protein levels of DPAGT1 (Fig. 3c, d). Intriguingly, BV6 significantly suppressed cell death induced by knockdown of DPAGT1 and non-toxic concentrations of TM (Fig. 3e). Together, this set of experiments shows that BV6 selectively protects from ER stress-induced apoptosis caused by pharmacological or genetic inhibition of N-linked protein glycosylation.

### BV6 counteracts TM-induced inhibition of protein glycosylation

**Fig. 4:**
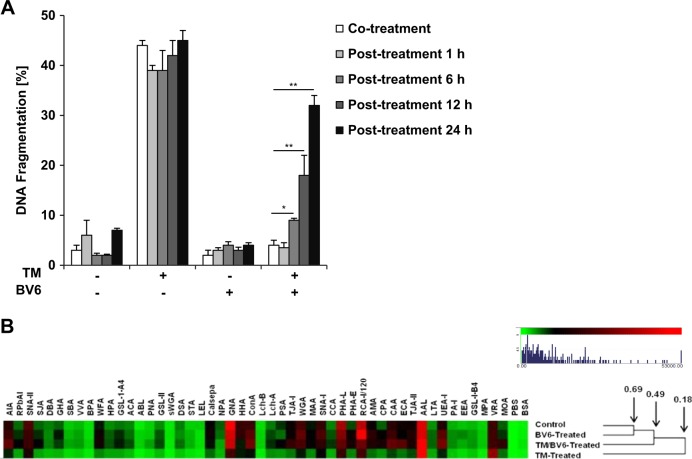
BV6 counteracts TM-induced inhibition of protein glycosylation. **a** SH-EP cells were cotreated for 72 h with 0.4 µg/ml TM and/or 4 µM BV6 or were treated for 72 h with 0.4 µg/ml TM and 4 µM BV6 were added at indicated time points. Apoptosis was determined by flow cytometric analysis of DNA fragmentation of PI-stained nuclei. Mean ± SEM of three independent experiments performed in triplicate is shown; *P < 0.05, **P < 0.001. **b** SH-EP cells were treated with 0.4 µg/ml TM and/or 4 µM BV6 for 3 h. Unsupervised hierarchical clustering of lectin microarray-binding intensities with membrane glycoprotein extracts from untreated (control), BV6-treated, TM/BV6-treated, and TM-treated cells. Averages of individual technical replicates were scaled (0–53,000RFU) and clustering was based on Euclidean distance and complete linkage method. ER, endoplasmic reticulum; TM, tunicamycin; PI, propidium iodide

To explore whether BV6 might impair the uptake of TM, we added BV6 simultaneously or, alternatively, at different time points after administration of TM. Notably, BV6 rescued TM-induced apoptosis when added up to 24 h after TM arguing against the possibility that BV6 impairs TM uptake (Fig. 4a). Nevertheless, BV6 progressively lost its ability to protect cells from TM-induced apoptosis in parallel with increasing the time gap between the administration of TM and the addition of BV6 (Fig. 4a). These data underline that BV6 counteracts TM’s effects upstream in the signaling pathway.

Therefore, we next addressed the question as to whether or not BV6 interferes with the TM-imposed inhibition of N-glycosylation as a very upstream effect of TM. To this end, we used lectin microarray profiling to investigate altered glycosylation of membrane or membrane-associated proteins^17^. SH-EP cells were treated with TM and/or BV6 and membrane proteins were extracted after 3 h of treatment. The same relative protein concentration was profiled for all samples, which allowed sample to sample comparison. Unsupervised clustering of normalized profile data was used to visualize data from lectin microarray^17^. This allowed detection of pattern differences between samples, indicating differential lectin binding and therefore differential glycosylation (Fig. 4b). This approach suggested that a broad variety of similar carbohydrate structures were present in each sample, including galactosylated and T-antigen structures (as indicated by binding of AIA and SNA-II), mannosylated structures (GNA, HHA, and ConA), complex carbohydrate structures (PHA-L and RCA-I), sialic acid (WGA and SNA-I), and fucose (AAL and UEA-I), respectively. The samples were divided at nodes according to the minimum similarities between their glycosylation patterns (Fig. 4b). Differences in lectin binding intensity as opposed to distribution were noted, suggesting that samples had similar patterns but different levels of glycosylation. Interestingly, the TM-treated samples notably stood apart from the three other samples. They only attained an 18% similarity to the control/BV6 and TM/BV6 clusters and displayed a lower lectin binding intensity when compared to control sample, indicating an overall reduced total glycosylation due to TM treatment, as expected (Fig. 4b). For example, significantly lower binding of TM-treated compared to control sample to the lectins NPA, GNA, Con A, Calsepa, and HHA implied a decrease in high mannose structures (Fig. 4b). Similarly, the reduced binding of the TM-treated sample to the lectins TJA-I, MAA, and WGA indicated a reduction in sialylation (Fig. 4b). A similar trend of reduced binding of the TM-treated sample was observed for *N*-acetylglucosamine-binding lectin (LEL), *N*-acetylgalactosamine-binding lectins (WFA), and T-antigen binding lectin (ACA) (Fig. 4b). In addition to this, a significant increase in VRA binding intensity for the TM-treated sample compared to all other samples was noted (Fig. 4b), suggesting an increase in α-linked galactose termination that was mirrored by the subtle increase in intensity at MOA. Importantly, the addition of BV6 countered the TM-induced reduction of lectin binding and the TM/BV6-treated sample profiles displayed more similarity to the untreated control and BV6-treated samples (Fig. 4b). BV6 alone had the least effect on protein glycosylation when compared to control sample and BV6-treated and control samples clustered together with 69% minimum similarity (Fig. 4b). Thus, these data show that the overall protein glycosylation level profile of the TM/BV6-treated sample was more similar to the control sample than the TM-treated sample suggesting that BV6 partially counteracts the effect of TM on total protein glycosylation.

### BV6 resolves the TM-induced UPR

**Fig. 5:**
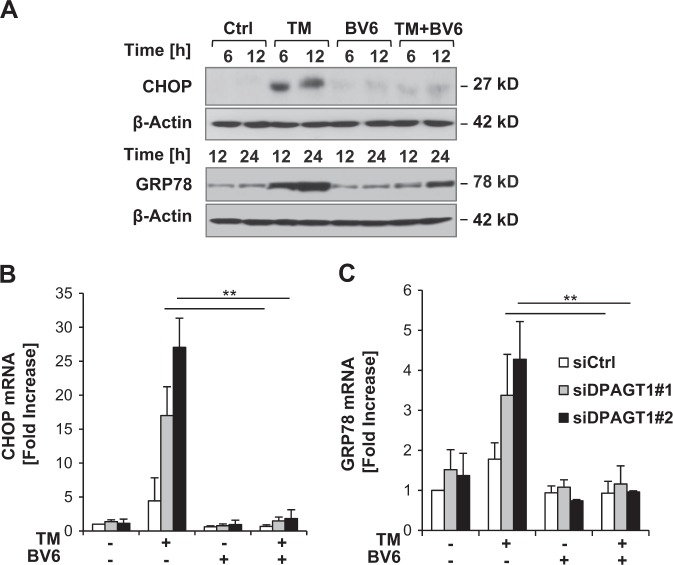
BV6 resolves the TM-induced UPR. **a** SH-EP cells were treated with 0.4 µg/ml TM and/or 4 µM BV6 for indicated time points and expression levels of CHOP and GRP78 were evaluated by Western blotting. **b**, **c** SH-EP cells were transiently transfected with siRNA sequence targeting DPAGT1 (siDPAGT1) or control siRNA (siCtrl) and treated with 0.05 µg/ml TM and/or 5 µM BV6. CHOP (B) and GRP78 (C) mRNA levels were analyzed after 4 h of treatment by quantitative RT-PCR and fold increase in mRNA levels are shown. Mean ± SEM values of three independent experiments are shown; **P < 0.001

To determine if BV6 resolves the UPR and restores ER homeostasis upon exposure to TM, we explored whether BV6 impairs upregulation of ER stress-dependent genes such as GRP78 and CHOP as markers of the impaired protein folding capacity of the ER^1^. Indeed, BV6 attenuated TM-stimulated upregulation of CHOP and GRP78 protein (Fig. 5a, Supplementary Figure 3). Furthermore, the addition of BV6 almost completely reduced the TM-stimulated increase in CHOP mRNA levels (Fig. 5b). Also, BV6 abolished the upregulation of GRP78 mRNA levels in TM-treated DPAGT1 knockdown cells (Fig. 5c). These findings show that BV6 prevents the induction of the UPR by TM.

### NF-κB contributes to BV6-mediated suppression of TM-stimulated UPR

**Fig. 6:**
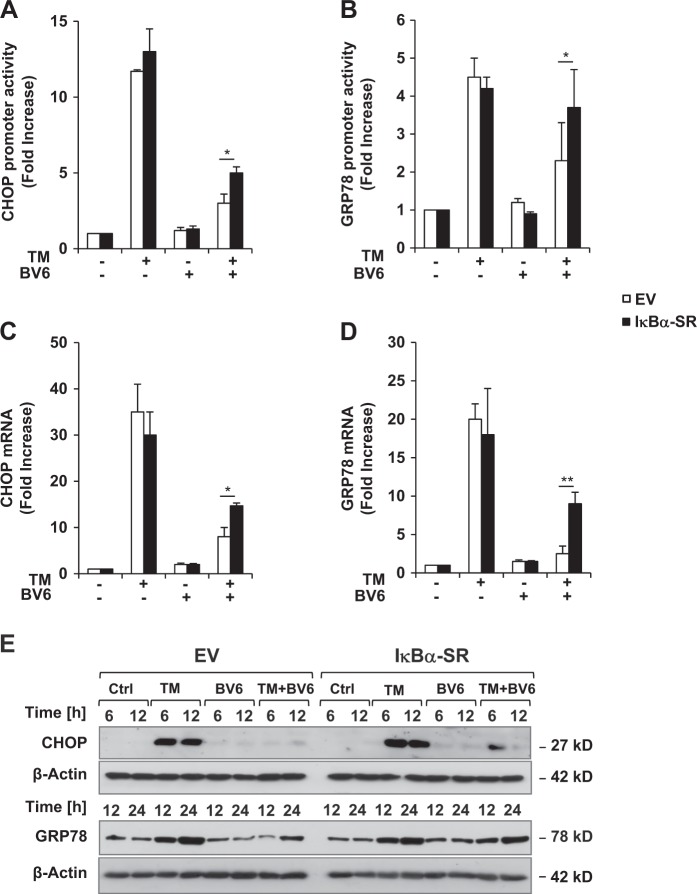
NF-κB contributes to BV6-mediated suppression of TM-stimulated UPR. **a**, **b** SH-EP cells stably expressing IκBα-SR or vector control were transfected with reporter firefly luciferase and Renilla luciferase vectors. Promoter activities of CHOP (**a**) or GRP78 (**b**) were measured 12 h after treatment with 0.4 µg/ml TM and/or 4 µM BV6. **c**, **d** SH-EP cells stably expressing IκBα-SR or vector control were treated for 12 h with 0.4 µg/ml TM and/or 4 µM BV6. Expression levels of CHOP (**c**) or GRP78 (**d**) were determined by quantitative RT-PCR and fold increase is shown. **e** SH-EP cells stably expressing IκBα-SR or vector control were treated for indicated time points with 0.4 µg/ml TM and/or 4 µM BV6. Expression levels of CHOP and GRP78 were analyzed by Western blotting. **a–d** Mean ± SEM of three independent experiments performed in triplicate are shown; *P < 0.05; **P < 0.001. UPR, unfolded protein response; GRP78, glucose-regulated protein 78; mRNA, messenger RNA; ER, endoplasmic reticulum; siRNA, small interfering RNA; TM, tunicamycin; PI, propidium iodide; RT-PCR, reverse transcription-PCR

Smac mimetics such as BV6 have been reported to activate NF-κB upon depletion of cIAP proteins^5,6^. Consistently, BV6 treatment or knockdown of cIAP1 and cIAP2 resulted in NF-κB activation in the presence and absence of TM, while single treatment with TM had little effect on NF-κB activation (Supplementary Figures 4A, 4B). Since NF-κB has been described to repress CHOP^18^, we then asked whether NF-κB is required for the BV6-conferred protection against TM-induced UPR and ER stress. To investigate the functional impact of NF-κB, we stably expressed dominant-negative superrepressor (IκBα-SR), which blocks canonical and non-canonical NF-κB activation^19^. Control experiments confirmed ectopic expression of IκBα-SR in IκBα-SR-overexpressing cells (Supplementary Figure 4C) as well as suppression of NF-κB activation, as documented by a lack of IκBα phosphorylation (Supplementary Figure 4C). NF-κB inhibition significantly, yet not completely, attenuated the BV6-imposed suppression of CHOP and GRP78 transcriptional activity in TM/BV6-cotreated cells (Fig. 6a, b). Consistently, mRNA levels of CHOP and GRP78 were significantly increased in IκBα-SR-overexpressing cells upon TM/BV6 cotreatment (Fig. 6c, d). Furthermore, NF-κB inhibition partially attenuated the BV6-mediated suppression of CHOP and GRP78 protein levels (Fig. 6e). As a consequence the BV6-imposed protection from TM-induced cell death was abolished in IκBα-SR-overexpressing cells (Supplementary Figure 4D). Together, these data demonstrate that NF-κB contributes to BV6-mediated suppression of TM-stimulated upregulation of CHOP and GRP78.

### BV6 suppresses TM-triggered ER stress response pathways

**Fig. 7:**
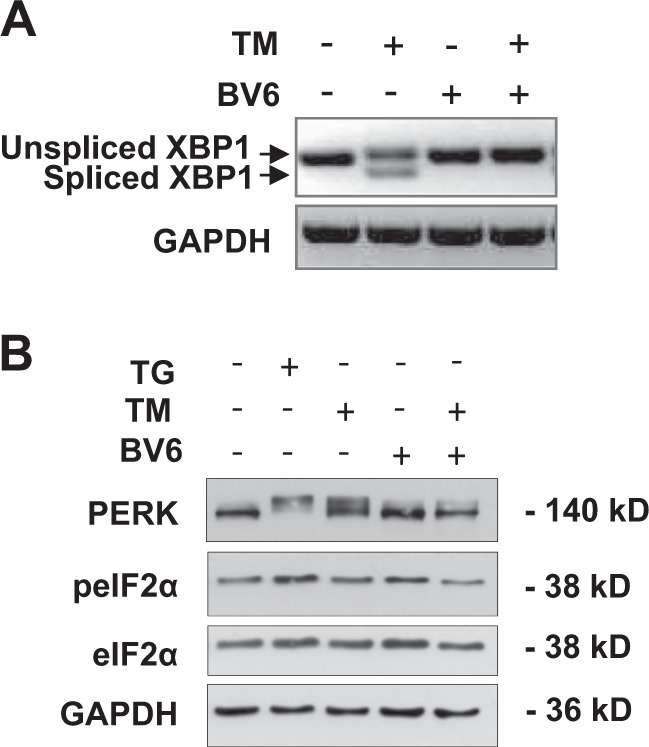
BV6 suppresses TM-triggered ER stress response pathways. **a** SH-EP cells were treated for 3 h with 0.4 µg/ml TM and/or 4 µM BV6. The splicing of XBP1 mRNA was analyzed by RT-PCR using XBP1 primers that detect both unspliced (289 bp) and spliced (263 bp) isoforms. **b** SH-EP cells were treated for 9 h with 0.4 µg/ml TM and/or 4 µM BV6, treatment with 10 µM TG was used as a positive control for phosphorylation of eIF2α. Expression of phospho-PERK (indicated by upward band shift), phospho-eIF2α and eIF2α was analyzed by Western blotting; GAPDH served as a loading control. ER, endoplasmic reticulum; TM, tunicamycin; PI, propidium iodide; RT-PCR, reverse transcription-PCR; PERK, protein kinase RNA-like ER kinase; GAPDH, glyceraldehyde-3-phosphate dehydrogenase

The UPR is transduced by several major ER stress pathways^2^. Therefore, we next investigated whether BV6 affects different branches of the ER stress response that are known to transmit the UPR signal^2^. As IRE1 is one of the major ER-resident transmembrane proteins that is activated upon induction of the UPR^2^, we determined activation of the IRE1-dependent ER stress pathway by analyzing XBP1 mRNA splicing as a marker of IRE1 activity. IRE1 catalyzes the excision of a 26-nucleotide intron out of the mRNA of XBP1, which leads to a shift in the coding reading frame and the generation of XBP1s, a transcription factor that controls genes involved in protein folding^2^. Importantly, PCR analysis showed that the addition of BV6 abolished the TM-stimulated splicing of XBP1 mRNA (Fig. 7a).

Furthermore, we investigated activation of the PERK-dependent ER stress pathway. As PERK undergoes autophosphorylation upon its activation^2^, we assessed PERK phosphorylation by Western blotting. Remarkably, while TM triggered an upward band shift of PERK, which marks its phosphorylation (Fig. 7b), the addition of BV6 to TM-treated cells reduced this upward band shift of PERK compared to cells treated with TM alone (Fig. 7b). Treatment with TG served as a positive control for PERK phosphorylation (Fig. 7b). Together, this set of experiments indicates that BV6 resolves TM-triggered ER stress response pathways, consistent with its ability to inhibit TM-induced apoptosis.

## Discussion

Since IAP proteins have been implicated in regulating the ER stress response^8–10^, the present study investigated the question as to whether or not small-molecule Smac mimetics that antagonize IAP proteins affect the UPR and ER stress-induced apoptosis. Here, we discover that Smac mimetics protect from TM-triggered apoptosis by resolving the UPR and ER stress. This protective function of Smac mimetics during TM-induced apoptosis is of broader relevance for structurally different Smac mimetics as well as for several cancer types. Moreover, the Smac mimetic BV6 not only transiently protects from TM-stimulated apoptotic cell death but also supports long-term survival.

Mechanistically, our study provides novel insights into the regulation of TM-imposed ER stress by Smac mimetics. We provide evidence showing that BV6 acts upstream in the ER stress pathway that is triggered by TM, as supported by several independent pieces of data. First, BV6 counteracts the TM-imposed inhibition of protein glycosylation as revealed by lectin profiling. Second, BV6 prevents the induction of the UPR upon TM treatment as shown by Smac mimetic-imposed inhibition of the accumulation of ER stress markers GRP78 and CHOP. Third, BV6 attenuates different arms of the ER stress response, as it inhibits TM-stimulated PERK phosphorylation as well as XPB1 splicing. Fourth, BV6 selectively protects from the distinct type of ER stress-induced apoptosis caused by pharmacological or genetic inhibition of N-linked protein glycosylation, but does not rescue from other modes of ER stress. Collectively, these pieces of evidence indicate that BV6 protects from TM-induced apoptosis by resolving the UPR and ER stress response.

Furthermore, our findings indicate that BV6-stimulated NF-κB activation contributes, at least in part, to BV6-imposed repression of the TM-induced ER stress response by suppressing TM-stimulated transcriptional activation of CHOP and GRP78. In the past, NF-κB has been described to confer resistance to ER stress by blocking CHOP^18^ or surface expression of GRP78 and subsequently Par-4-mediated apoptosis^20^.

The most informative finding of the current work is the discovery of a protective function of Smac mimetics during TM-induced ER stress. Smac mimetics are small-molecule inhibitors of IAP proteins which are expressed at high levels in various cancer entities. Since IAP proteins play a critical role in blocking cell death and promoting survival of cancer cells, Smac mimetics are considered as promising novel cancer therapeutics. By antagonizing IAP proteins such as cIAP proteins and XIAP, Smac mimetics have been shown to trigger apoptosis as single agents or in combination in a large variety of human cancers^21^. For example, we previously reported that Smac mimetics such as BV6 enhance apoptosis in response to different cell death stimuli, including death receptor ligands^22–29^, chemotherapy^30–32^, or γ-irradiation^33–35^. In addition, we recently found that under certain conditions BV6 also exerts non-apoptotic functions and promotes migration, invasion, and differentiation of cancer cells in an NF-κB-dependent manner^36,37^. Together, these studies show that Smac mimetics are involved in the regulation of various biological processes and exert much more complex functions than initially assumed. A number of Smac mimetics, including those that have been tested in the present study, are currently being evaluated in early clinical trials in different cancers^38^. By providing new insights into the regulation of cellular stress responses by Smac mimetics, our study potentially has important implications for aberrant ER stress responses that are involved in the pathogenesis of many human diseases and for the use of Smac mimetics as cancer therapeutics.

## Materials and methods

### Cell culture and chemicals

Cell lines were obtained from the American Type Culture Collection (Manassas, VA, USA) and maintained in MEMα, RPMI 1640 or Dulbecco’s modified Eagle's medium medium (Invitrogen, Karlsruhe, Germany), supplemented with 10% fetal calf serum and 25 mM 4-(2-hydroxyethyl)-1-piperazineethanesulfonic acid (HEPES) (both from Biochrom, Berlin, Germany), 1 mM l-glutamine and 1% penicillin/streptomycin (both from Invitrogen), as described previously^39^. The bivalent Smac mimetic BV6 has previously been described^5^ and was kindly provided by Genentech Inc. (South San Francisco, CA, USA). IAP inhibitor 2 resembles the compound 11 described by Oost et al.^40^ and IAP inhibitor 3 was described by Chao et al.^41^ and were kindly provided by Idun Pharmaceuticals now Pfizer (Groton, CT, USA). Birinapant was obtained from Selleck Chemicals (Newmarket, UK). zVAD.fmk was purchased from Bachem (Heidelberg, Germany), TM from AppliChem (Darmstadt, Germany), BFA from Cell Signaling (Beverly, MA), 2-DG, 4-hydroxytamoxifen, cithiothreitol, and TG from Sigma (Deisenhofen, Germany) and all other chemicals from Sigma (Deisenhofen, Germany), unless indicated otherwise. Bortezomib was obtained from Jansen-Cilag (Neuss, Germany). The Mem-PER Eukaryotic Membrane Protein Extraction Reagent Kit, protease inhibitor cocktail (EDTA-free), and Alexa Fluor^®^ 555 NHS ester were purchased from Thermo Fisher Scientific Inc. (Dublin, Ireland). Centrifugal Ultracel^®^ low binding regenerated cellulose filters (0.5 ml, 3 kDa molecular weight cut-off (MWCO)) were obtained from Merck Millipore (Cork, Ireland), and the Nexterion^®^ slide H microarray slides from Schott AG (Mainz, Germany).

### Determination of apoptosis, cell viability, and colony formation

Apoptosis was determined by analysis of DNA fragmentation of propidium iodide (PI)-stained nuclei using flow cytometry (FACSCanto II, BD Biosciences, Heidelberg, Germany) as described previously^39^. Cell death was determined by fluorescence-based microscopic analysis of PI uptake using Hoechst 33342 and PI double-staining and ImageXpress Micro XLS Widefield High-Content Analysis System and MetaXpress^®^ software according to the manufacturer’s instructions (Molecular Devices, Sunnyvale, CA, USA). Cell viability was assessed by 3-(4,5-dimethylthiazol-2-yl)-2,5-diphenyltetrazolium bromide (MTT) assay according to the manufacturer’s instructions (Roche Diagnostics, Mannheim, Germany). For colony formation assay, cells were seeded in a 6-well plate (100 cells per well) and treated with TM and/or BV6 for 48 h, placed in drug-free medium, and cultured for 14 days. Surviving colonies were stained with crystal violet solution (0.75% crystal violet containing 50% ethanol, 0.25% NaCl, and 1.57% formaldehyde).

### Western blot analysis

Western blot analysis was performed as described previously^39^ using the following antibodies: mouse anti-caspase-8 (Alexis Biochemicals, Grünberg, Germany), mouse rabbit anti-caspase-3, rabbit anti-caspase-9, mouse anti-CHOP, rabbit anti-GRP78, rabbit anti-PERK (all from Cell Signaling, Beverly, MA, USA), rabbit anti-DPAGT1 antibody (Abcam, Cambridge, MA, USA). Mouse anti-β-actin, mouse anti-vinculin (Sigma-Aldrich), or mouse anti-glyceraldehyde 3-phosphate dehydrogenase (HyTest, Turku, Finland) were used as loading controls. Goat anti-mouse immunoglobulin G (IgG), donkey anti-goat IgG, and goat anti-rabbit IgG conjugated to horseradish peroxidase (Santa Cruz Biotechnology, Santa Cruz, CA, USA) were used as secondary antibodies. Enhanced chemiluminescence was used for detection (Amersham Bioscience, Freiburg, Germany). Representative blots of at least two independent experiments are shown.

### Retroviral transduction and RNA interference-mediated gene silencing

Overexpression of the dominant-negative IκBα-SR was performed by retroviral transduction using IκBα (S32; 36A) and the pCFG5-IEGZ retroviral vector system as previously described^42^. HEK293T producer cells were transfected with 7.5 µg pGIPZ-shRNAmir vector, 12.5 µg pCMV-dR8.91, and 1 µg pMD2.G using calcium phosphate transfection. For transient knockdown by siRNA, cells were reversely transfected with 5 nM SilencerSelect siRNA (Invitrogen) control siRNA (# 4390843) or targeting siRNAs (s4242 and s4244 for DPAGT1, s1449 for cIAP1, s1452 for cIAP2) using Lipofectamine RNAi Max (Invitrogen) and OptiMEM (Life Technologies).

### Quantitative RT-PCR

Total RNA was extracted using peqGOLD Total RNA kit from Peqlab Biotechnologie GmbH (Erlangen, Germany) according to the manufacturer’s instructions. Two micrograms of total RNA were used to synthetize the corresponding complementary DNA using RevertAid H Minus First Strand cDNA Synthesis kit (MBI Fermentas GmbH, St. Leon-Rot, Germany). To quantify gene expression levels, SYBR-Green-based quantitative reverse transcription-PCR (RT-qPCR) was performed using the 7900HT Fast Real-Time PCR System from Applied Biosystems (Darmstadt, Germany). Data were normalized on 28S ribosomal RNA expression as the reference gene. Primers are listed in Supplementary Table 1. Melting curves were plotted to verify the specificity of the amplified products. All determinations were performed in triplicate. The relative expression of the target gene transcript and reference gene transcript was calculated as ΔΔCt.

### Detection of XBP1 splicing

The splicing of XBP1 mRNA was analyzed by RT-PCR using primers specific for *XBP1*, which detect both unspliced and spliced isoforms (Supplementary Table 1). PCR products were separated by electrophoresis on 3% agarose gels and visualized by ethidium bromide staining.

### Lectin microarray glycoprofiling

Lectin microarray profiling was done essentially as previously described^17^.

### Protein preparation and labeling

Membrane and membrane-associated proteins were extracted from cells using the Mem-PER Eukaryotic Membrane Protein Extraction Reagent kit following the manufacturer’s instructions, except with the inclusion of protease inhibitor cocktail (1/100, v/v) at every step. Protein extracts (1–2 mg) were directly labeled with Alexa Fluor^®^ 555 NHS ester in a final 0.1 M solution of sodium bicarbonate, pH 8.3, for 2 h at room temperature in the dark and all subsequent steps were carried out with limited light exposure. Excess dye was removed from labeled membrane protein by 3 kDa MWCO centrifugal filtration with seven washes of 0.5 ml phosphate-buffered saline (14,000 x *g*, 20 min per wash). Final protein content and labelling efficiency for each sample were calculated according to the manufacturer’s instructions using absorbance at 280 and 555 nm.

### Lectin microarray construction and incubation

A panel of 50 lectins from plant, bacterial, and fungal origin (Supplementary Table 2) was used to construct lectin microarrays on Nexterion^®^ slide H microarray slides, essentially as previously described^17^. Lectin microarray incubations were carried out as previously described^17^ in the dark with the addition of a labeled asialofetuin included in one subarray of each microarray slide to verify lectin print and performance. Titration of the labeled samples was first carried out to determine the optimum incubation concentration, followed by triplicate profiling and analysis. Each labeled sample (at 2.5 μg/ml dilution in TBST) was incubated on the lectin microarray slides in triplicate and incubated in a rotating oven at 23 °C for 1 h. Slides were washed three times in Tris-buffered saline supplemented with Ca^2+^ and Mg^2+^ ions (TBS; 20 mM Tris-HCl, 100 mM NaCl, 1 mM CaCl_2_, 1 mM MgCl_2_, pH 7.2) with 0.05% Tween-20 (TBS-T) and once in TBS. The slides were then centrifuged dry (450 x *g*, 5 min) and scanned using an Agilent G2505B microarray scanner (532 nm channel, 90% PMT; Agilent, Cork Ireland).

### Data extraction and analysis

Raw intensity values were extracted from microarray images using GenePix Pro 6.1.0.4 (Molecular Devices, Sunnyvale, CA, USA) and exported to Microsoft Excel for data analysis essentially as previously described^17^. Unsupervised hierarchical clustering of lectin binding data was performed with Hierarchical Clustering Explorer v3.0 to explore similarities in binding intensities between SH-EP cells and cells treated with TM and/or BV6 (HCE; http://www.cs.umd.edu/hcil/hce/hce3.html). The means of three normalized individual technical replicates were scaled over the dynamic range of the lectin array (0–53,000 relative fluorescence units (RFU)) and clustered with the following parameters: no pre-filtering, Euclidean distance, and complete linkage.

### Statistical analysis

Statistical significance was assessed by Student’s *t* test (two-tailed distribution, two-sample, unequal variance), *P < 0.05, **P < 0.001.

## Supplementary information

Supplemental Material
