# Correction: Smac mimetic suppresses tunicamycin-induced apoptosis via resolution of ER stress

**DOI:** 10.1038/s41419-020-02991-z

**Published:** 2020-09-25

**Authors:** Behnaz Ahangarian Abhari, Nicole McCarthy, Marie Le Berre, Michelle Kilcoyne, Lokesh Joshi, Patrizia Agostinis, Simone Fulda

**Affiliations:** 1 Institute for Experimental Cancer Research in Pediatrics, Goethe-University Frankfurt, Komturstrasse 3a, 60528 Frankfurt, Germany; 2 Glycoscience Group, National University of Ireland, Galway, Ireland; 3 Cell Death Research and Therapy Unit, Department of Cellular and Molecular Medicine, KU Leuven, 3000 Leuven, Belgium; 4 German Cancer Consortium (DKTK), Partner Site Frankfurt, Germany; 5 German Cancer Research Center (DKFZ), Heidelberg, Germany

**Keywords:** Targeted therapies, Cancer therapeutic resistance

Correction to: *Cell Death & Disease*

10.1038/s41419-019-1381-z

published online 15 February 2019

Since online publication of this article, the authors noticed that Fig. 3b does not show the correct graph for Bortezomib. The corrected graph for Fig. 3b is provided below. This unintentional mistake does not alter the conclusions of the study. The authors apologise for any inconvenience caused.

**Fig. 3:**
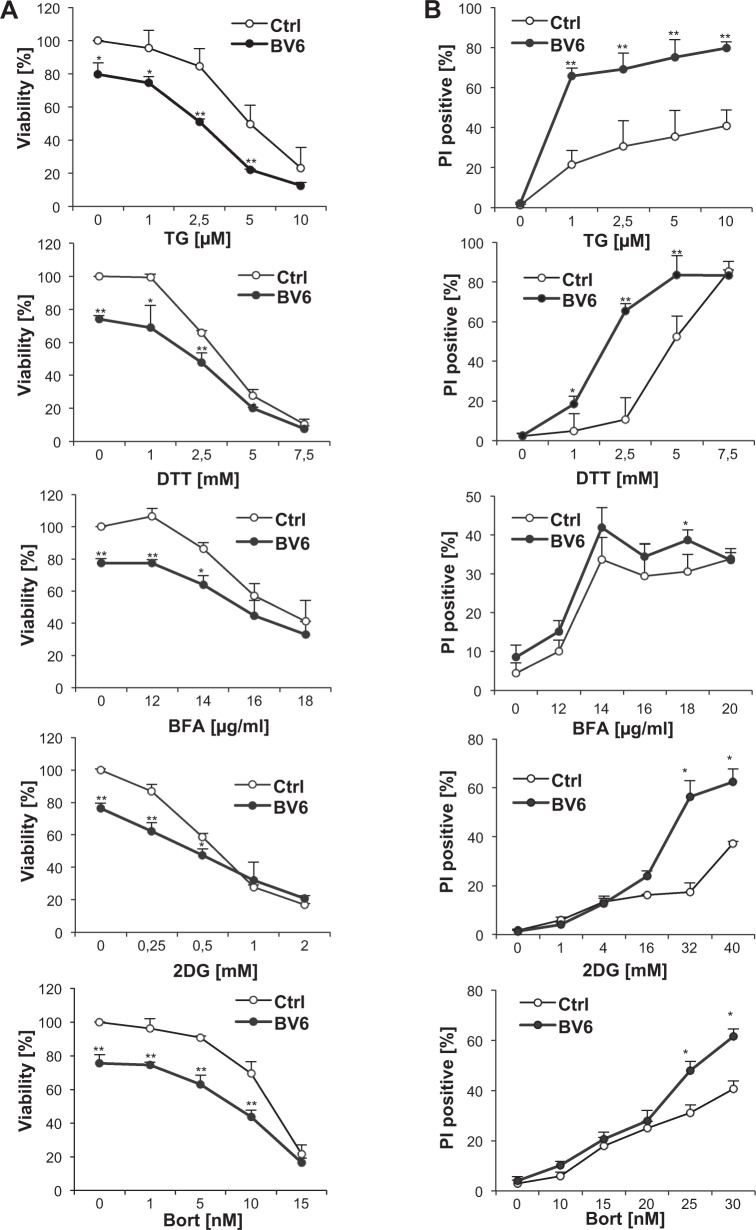
BV6 selectively protects from ER stress-induced apoptosis caused by inhibition of N-linked protein glycosylation.

