# Supplementary material for: Smac mimetic suppresses tunicamycin-induced apoptosis via resolution of ER stress
Source: Cell Death Dis. 2019 Feb 15;10(3):155. doi: 10.1038/s41419-019-1381-z (PMC6377606; doi:10.1038/s41419-019-1381-z)
Supplement: Supplementary file 1 — Supplemental Material [file 41419_2019_1381_MOESM1_ESM.pdf]

sFig. 1

A

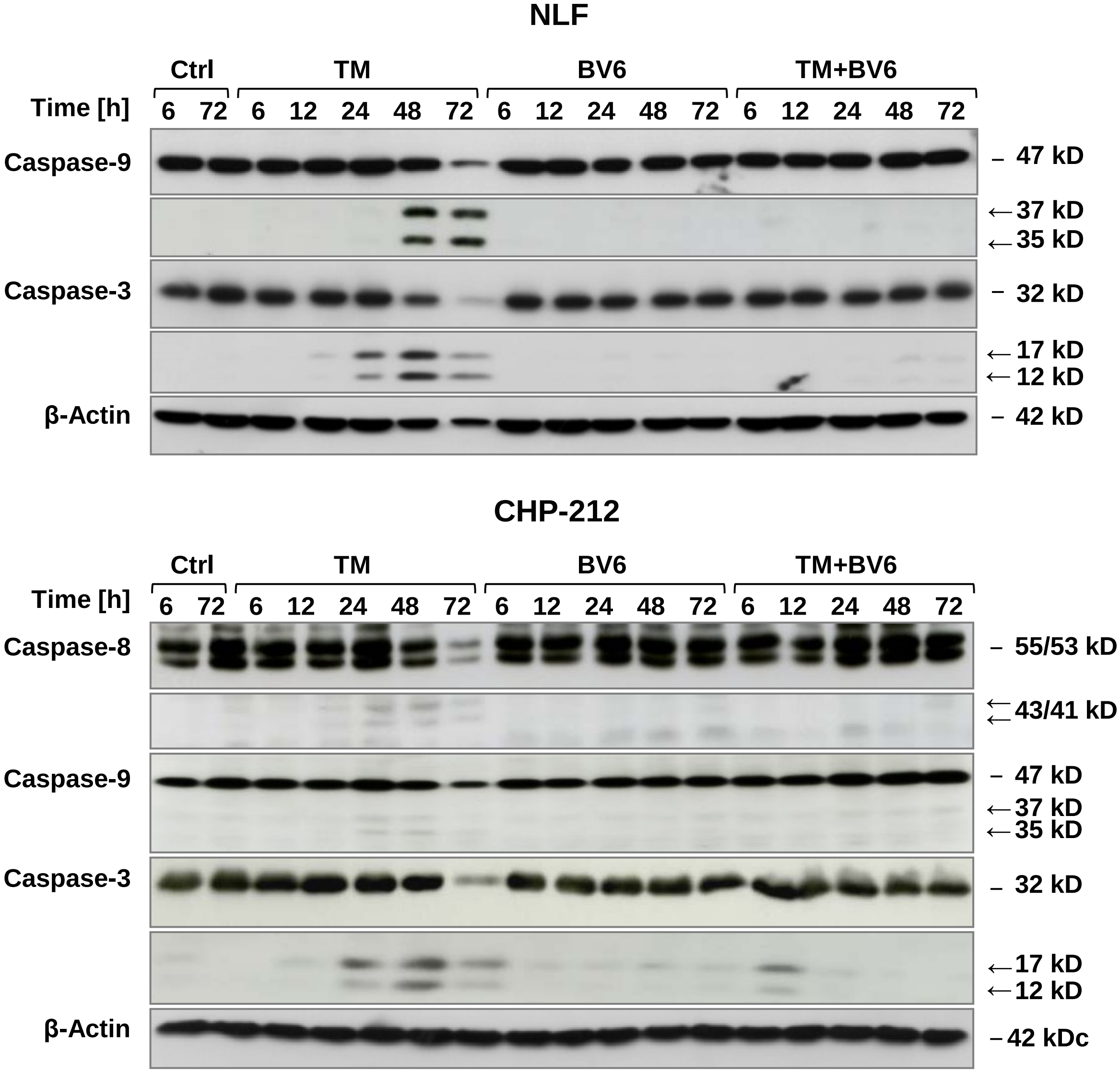
NLF
Ctrl
TM
BV6
TM+BV6
Time [h]
6 72 6 12 24 48 72 6 12 24 48 72 6 12 24 48 72
Caspase-9
47 kD
37 kD
35 kD
Caspase-3
32 kD
17 kD
12 kD
β-Actin
42 kD
CHP-212
Ctrl
TM
BV6
TM+BV6
Time [h]
6 72 6 12 24 48 72 6 12 24 48 72 6 12 24 48 72
Caspase-8
55/53 kD
43/41 kD
Caspase-9
47 kD
37 kD
35 kD
Caspase-3
32 kD
17 kD
12 kD
β-Actin
42 kDc

B

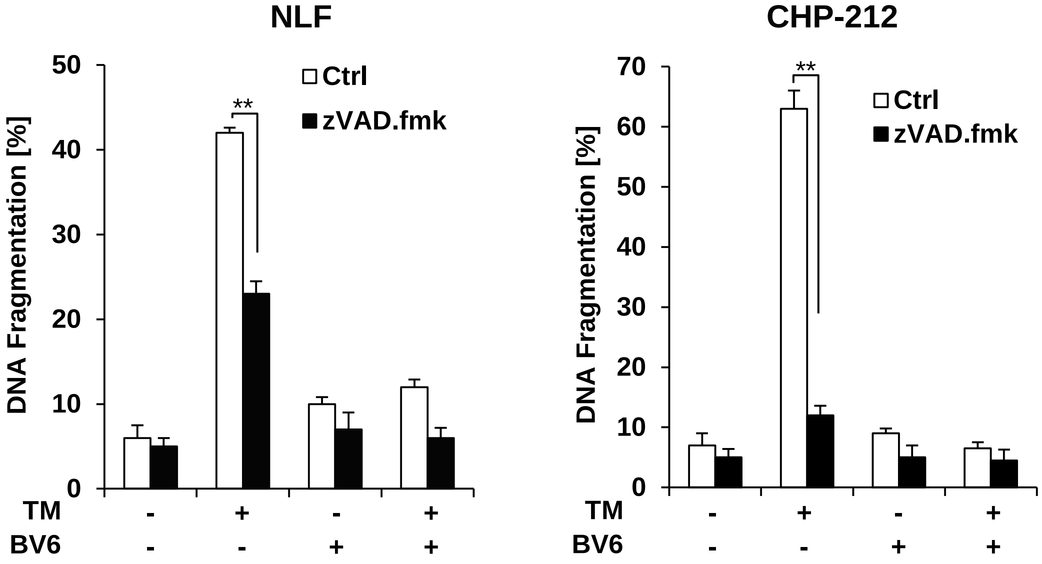
NLF
DNA Fragmentation [%]
Ctrl
zVAD.fmk
**
TM - + - +
BV6 - - + +
CHP-212
DNA Fragmentation [%]
Ctrl
zVAD.fmk
**
TM - + - +
BV6 - - + +

sFig. 2

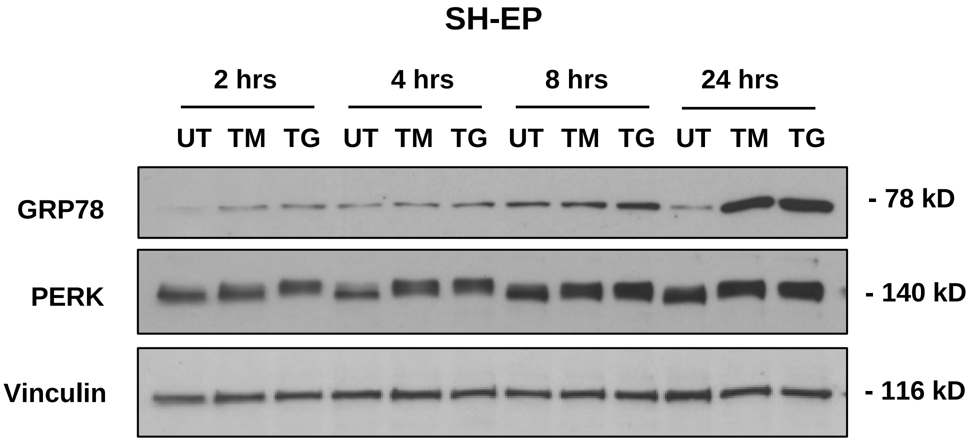

SH-EP
2 hrs
4 hrs
8 hrs
24 hrs
UT TM TG UT TM TG UT TM TG UT TM TG
GRP78
PERK
Vinculin
- 78 kD
- 140 kD
- 116 kD

sFig. 3

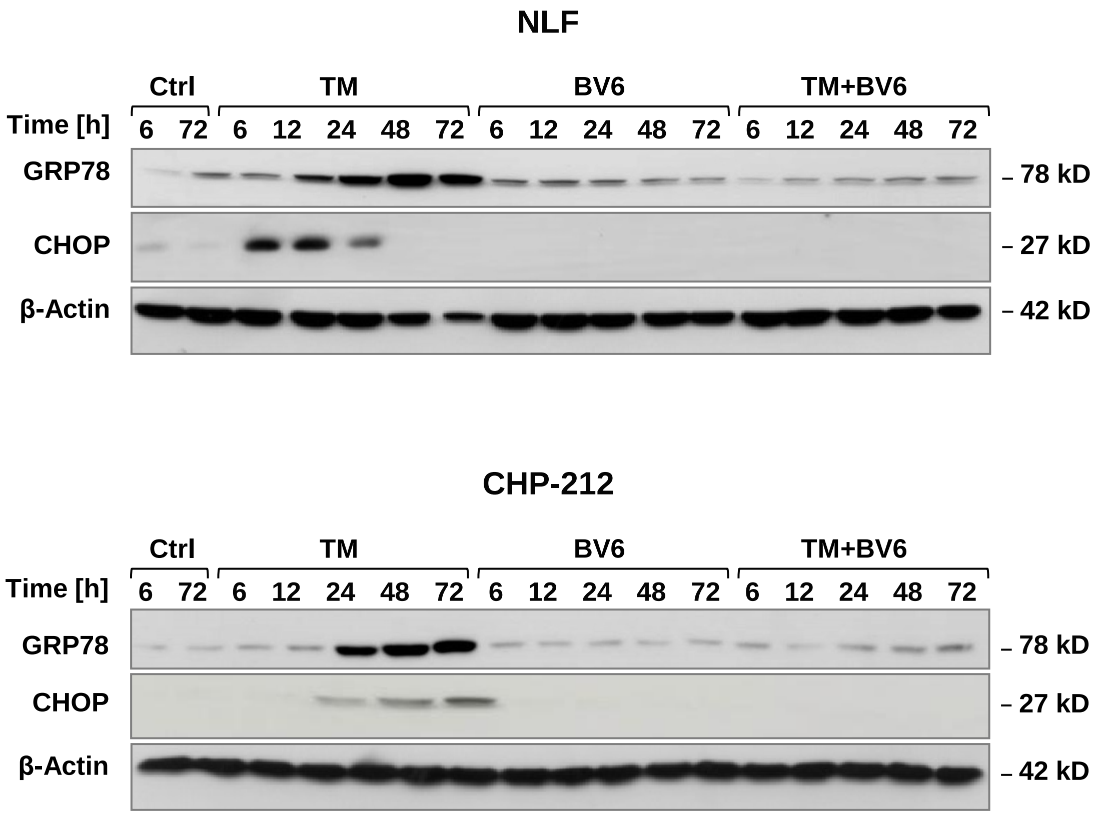
NLF
Ctrl
TM
BV6
TM+BV6
Time [h]
6 72 6 12 24 48 72 6 12 24 48 72 6 12 24 48 72
GRP78
CHOP
β-Actin
78 kD
27 kD
42 kD
CHP-212
Ctrl
TM
BV6
TM+BV6
Time [h]
6 72 6 12 24 48 72 6 12 24 48 72 6 12 24 48 72
GRP78
CHOP
β-Actin
78 kD
27 kD
42 kD

sFig. 4

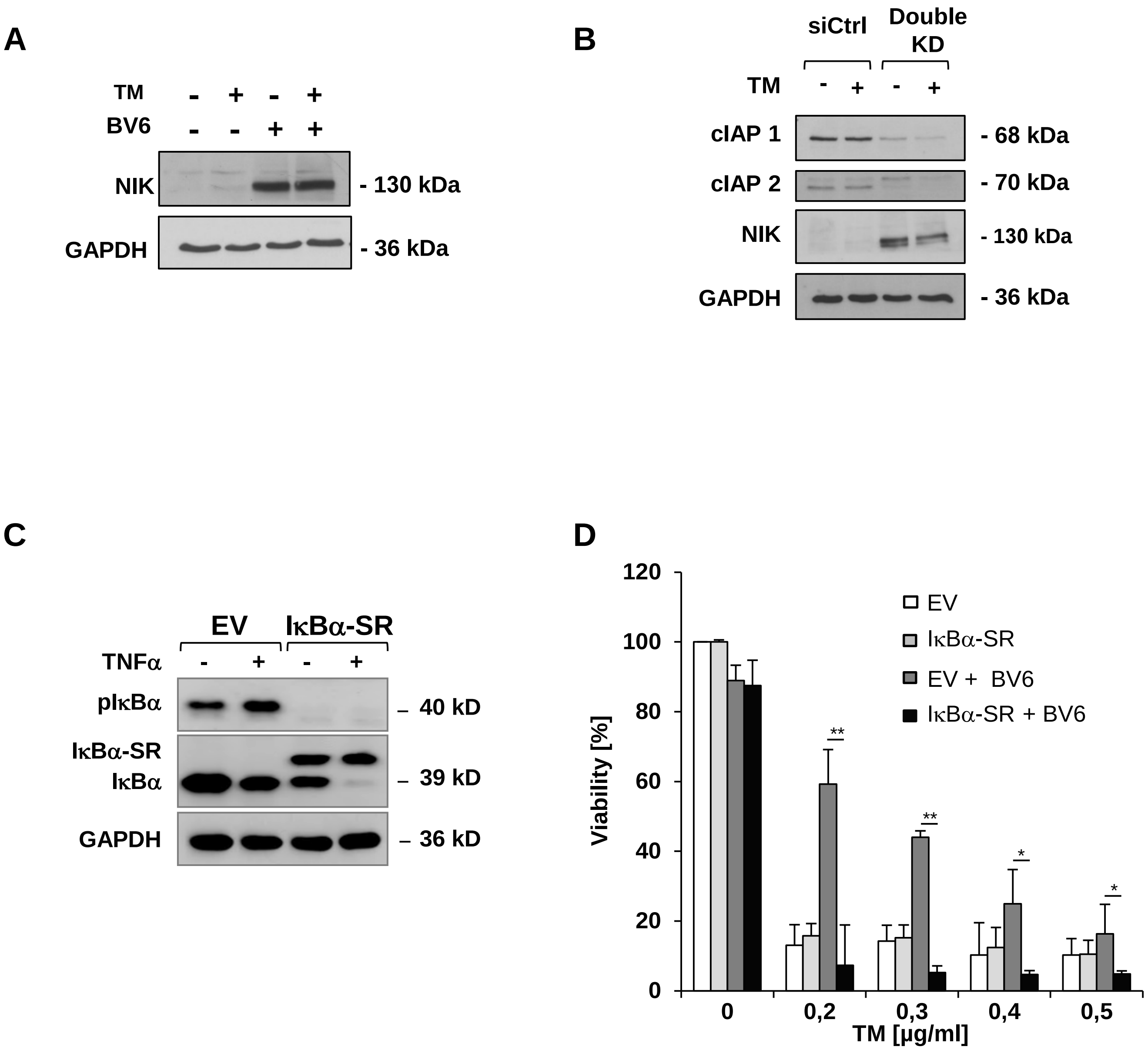
A
TM - + - +
BV6 - - + +
NIK
- 130 kDa
GAPDH
- 36 kDa
B
siCtrl
Double KD
TM - + - +
cIAP 1
- 68 kDa
cIAP 2
- 70 kDa
NIK
- 130 kDa
GAPDH
- 36 kDa
C
EV
IκBα-SR
TNFα - + - +
pIκBα
– 40 kD
IκBα-SR
IκBα
– 39 kD
GAPDH
– 36 kD
D
EV
IκBα-SR
EV + BV6
IκBα-SR + BV6
Viability [%]
120
100
80
60
40
20
0
**
**
*
*
0
0,2
0,3
0,4
0,5
TM [µg/ml]

Suppl. Table 1. Primer sequences

Primer	Sequence (5´- 3´)
h28S_forward	ttgaaaatccgggggagag
h28S_reverse	acattgttccaacatgccag
hCHOP_forward	aaggcactgagcgtatcatgt
hCHOP_reverse	tgaagatacacttccttcttgaacac
hDPAGT1_forward	ttccccttcctgaactgct
hDPAGT1_reverse	cacctatcagggccacaaat
hGRP78_forward	cagcctggcgacaagagt
hGRP78_reverse	ccttgggcagtattggattc
hXBP1_forward	ttacgagagaaaactcatggcc
hXBP1_reverse	gggtccaagttgtccagaatgc

Suppl. Table 2. Lectin microarray list

Lectin	Organism	Common name	Type	Print in	Specificity
AIA, Jacalin	*Artocarpus integrifolia*	Jack fruit lectin	Plant	Gal	Gal (sialylation tolerant)
RPbAI	*Robinia pseudoacacia*	Black locust lectin	Plant	Gal	Gal, GalNAc
SNA-II	*Sambucus nigra*	Sambucus lectin-II	Plant	Gal	Gal/GalNAc
SJA	*Sophora japonica*	Pagoda tree lectin	Plant	Gal	β-GalNAc
DBA	*Dolichos biflorus*	Horse gram lectin	Plant	Gal	GalNAc
GHA	*Glechoma hederacea*	Ground ivy lectin	Plant	Gal	GalNAc
SBA	*Glycine max*	Soy bean lectin	Plant	Gal	GalNAc
VVA	*Vicia villosa*	Hairy vetch lectin	Plant	Gal	GalNAc
BPA	*Bauhinia purpurea*	Camel's foot tree lectin	Plant	Gal	GalNAc/Gal
WFA	*Wisteria floribunda*	Japanese wisteria lectin	Plant	Gal	GalNAc/sulfated GalNAc
HPA	*Helix pomatia*	Garden snail lectin	Animal	Gal	α-GalNAc
GSL-I-A4	*Griffonia simplicifolia*	Griffonia lectin-I A4	Plant	Gal	GalNAc
ACA	*Amaranthus caudatus*	Amaranthin	Plant	Lac	Sialylated/Gal-β-(1,3)-GalNAc
ABL	*Agaricus bisporus*	Edible mushroom lectin	Fungus	Lac	Gal-β-(1,3)-GalNAc, GlcNAc
PNA	*Arachis hypogaea*	Peanut lectin	Plant	Gal	Gal-β-(1,3)-GalNAc
GSL-II	*Griffonia simplicifolia*	Griffonia/Bandeiraea lectin-II	Plant	GlcNAc	GlcNAc
sWGA	*Triticum vulgaris*	Succinyl WGA	Plant	GlcNAc	GlcNAc
DSA	*Datura stramonium*	Jimson weed lectin	Plant	GlcNAc	GlcNAc
STA	*Solanum tuberosum*	Potato lectin	Plant	GlcNAc	GlcNAc oligomers
LEL	*Lycopersicum eculentum*	Tomato lectin	Plant	GlcNAc	GlcNAc-β-(1,4)-GlcNAc
Calsepa	*Calystegia sepium*	Bindweed lectin	Plant	Man	Man/Maltose
NPA	*Narcissus pseudonarcissus*	Daffodil lectin	Plant	Man	α-(1,6)-Man
GNA	*Galanthus nivalis*	Snowdrop lectin	Plant	Man	Man-α(1,3)-
HHA	*Hippeastrum hybrid*	Amaryllis agglutinin	Plant	Man	Man-α(1,3)-Man-α(1,6)-
ConA	*Canavalia ensiformis*	Jack bean lectin	Plant	Man	Man, Glc, GlcNAc
Lch-B	*Lens culinaris*	Lentil isolectin B	Plant	Man	Man, fucose dependent
Lch-A	*Lens culinaris*	Lentil isolectin A	Plant	Man	Man, fucose dependent
PSA	*Pisum sativum*	Pea lectin	Plant	Man	Man, fucose dependent
TJA-I	*Trichosanthes japonica*	TJA One	Plant	Lac	Sialic acid-α-(2,6)-Gal(NAc)
WGA	*Triticum vulgaris*	Wheat germ agglutinin	Plant	GlcNAc	NeuAc/GlcNAc
MAA	*Maackia amurensis*	Maackia agglutinin	Plant	Lac	Sialic acid-α-(2,3)-Gal(NAc)
SNA-I	*Sambucus nigra*	Sambucus lectin-I	Plant	Lac	Sialic acid-α-(2,6)-Gal(NAc)
CCA	*Cancer antennarius*	California crab	Animal	Lac	9-*O*-acetyl-sialic acid
PHA-L	*Phaseolus vulgaris*	Kidney bean leukoagglutinin	Plant	Lac	tri-/tetra-antennary β-Gal/Gal-β-(1,4)-GlcNAc
PHA-E	*Phaseolus vulgaris*	Kidney bean erythroagglutinin	Plant	Gal	biantennary, bisecting GlcNAc,β-Gal/Gal-β-(1,4-)GlcNAc
RCA-I/120	*Ricinus communis*	Castor bean lectin I	Plant	Gal	Gal-β-(1,4)-GlcNAc
AMA	*Arum maculatum*	Lords and Ladies agglutinin	Plant	Lac	Gal-β-(1,4)-GlcNAc
CPA	*Cicer arietinum*	Chickpea lectin	Plant	Lac	Complex glycopeptides
CAA	*Caragana arborescens*	Pea tree lectin	Plant	Lac	Gal-β-(1,4)-GlcNAc
ECA	*Erythrina cristagalli*	Cocks comb/coral tree lectin	Plant	Lac	Gal-β-(1,4)-GlcNAc oligomers
TJA-II	*Trichosanthes japonica*	TJA Two	Plant	Gal	Fuc-α(1,2)Gal(NAc)-β(1,4)
AAL	*Aleuria aurantia*	Orange peel fungus lectin	Fungus	Fuc	α-(1,6)-linked Fuc
LTA	*Lotus tetragonolobus*	Lotus lectin	Plant	Fuc	α-(1,3)-linked Fuc
UEA-I	*Ulex europaeus*	Gorse lectin-I	Plant	Fuc	α-(1,2)-linked Fuc
PA-I	*Pseudomonas aeruginosa*	Pseudomonas lectin	Bacteria	Gal	Gal, Gal derivatives
EEA	*Euonymous europaeus*	Spindle tree lectin	Plant	Gal	α-Gal
GSL-I-B4	*Griffonia simplicifolia*	Griffonia/Bandeiraea lectin-I	Plant	Gal	α-Gal
MPA	*Maclura pomifera*	Osage orange lectin	Plant	Gal	α-Gal
VRA	*Vigna radiata*	Mung Bean Lectin	Plant	Gal	α-Gal
MOA	*Marasmius oreades*	Fairy ring mushroom lectin	Fungus	Gal	α-Gal
**PBS**	**N/A**	**N/A**	**N/A**	NS	N/A
**BSA**	**N/A**	**N/A**	**Animal**	NS	N/A

Supplemental Figure legends

Suppl. Figure 1. BV6 inhibits TM-induced caspase activation and caspase-dependent apoptosis in neuroblastoma cells.

(A) NLF and CHP-212 cells were treated for indicated times with TM (NLF: 0.1 µg/ml; CHP-212: 0.2 µg/ml) and/or 5 µM BV6. Caspase activation was analyzed by Western blotting, cleavage fragments are indicated by arrows.

(B) NLF and CHP-212 cells were treated for 72 hours with TM (NLF: 0.1 µg/ml; CHP-212: 0.2 µg/ml) and/or 5 µM BV6 in the presence or absence of 20 µM zVAD.fmk. Apoptosis was determined by flow cytometric analysis of DNA fragmentation of PI-stained nuclei. Mean+SEM of three independent experiments performed in triplicate are shown; $^{**}P<0.01$.

Suppl. Figure 2. Effect of TM and TG on the UPR and ER stress.

SH-EP cells were left untreated (UT) or were treated for indicated times with 0.4 µg/ml TM and/or 2.5 µM TG. Expression of GRP78 and phospho-PERK (indicated by upward band shift) was analyzed by Western blotting; Vinculin served as loading control.

Suppl. Figure 3. BV6 resolves TM-induced UPR.

NLF and CHP-212 neuroblastoma cells were treated for indicated times with TM (NLF: 0.1 µg/ml; CHP-212: 0.2 µg/ml) and/or 5 µM BV6. Expression levels of CHOP and GRP78 were evaluated by Western blotting. Expression of β-actin served as loading control.

Suppl. Figure 4. Involvement of NF-κB signaling.

(A) SH-EP cells were treated for six hours with 0.4 µg/ml TM and/or 4 µM BV6. Expression of NIK was analyzed by Western blotting, GAPDH served as loading control.

(B) SH-EP cells were transiently transfected with siRNAs against cIAP1 and cIAP2 or with control siRNA and were treated with 0.4 µg/ml TM. Expression of NIK was analyzed by Western blotting, GAPDH served as loading control.

(C) SH-EP cells stably expressing IκBα-SR or empty vector (EV) were treated with 10 ng/ml TNFα for one hour. Expression of IκBα, IκBα-SR and phospho-IκBα was assessed by Western blotting, GAPDH served as loading control.

(D) SH-EP cells stably expressing IκBα-SR or vector control were treated for 72 hours with 0.4 µg/ml TM and/or 4 µM BV6. Cell viability was assessed by MTT assay and is expressed as the percentage of untreated controls. Mean + SEM of three independent experiments performed in triplicate are shown; *P<0.05; **P<0.001.

Suppl. Table 1. Primer sequences.

Suppl. Table 2. Lectin microarray list.
